# The incremental benefit of EUS for identifying unresectable disease among adults with pancreatic adenocarcinoma: A meta-analysis

**DOI:** 10.1371/journal.pone.0173687

**Published:** 2017-03-20

**Authors:** Paul D. James, Zhao Wu Meng, Mei Zhang, Paul J. Belletrutti, Rachid Mohamed, William Ghali, Derek J. Roberts, Guillaume Martel, Steven J. Heitman

**Affiliations:** 1 Department of Medicine and the Ottawa Hospital Research Institute, Department of Medicine, University of Ottawa, Ottawa, Canada; 2 Department of Community Health Sciences, University of Calgary, Calgary, Canada; 3 Department of Medicine, University of Calgary, Calgary, Canada; 4 Calgary Research and Education in Advanced Therapeutic Endoscopy (CREATE), Calgary, Canada; 5 Department of Surgery, University of Calgary, Calgary, Canada; 6 Department of Surgery, University of Ottawa, Ottawa, Canada; Hvidovre Hospital, DENMARK

## Abstract

**Background and study aims:**

It is unclear to what extent EUS influences the surgical management of patients with pancreatic adenocarcinoma. This systematic review sought to determine if EUS evaluation improves the identification of unresectable disease among adults with pancreatic adenocarcinoma.

**Patients and methods:**

We searched MEDLINE, EMBASE, bibliographies of included articles and conference proceedings for studies reporting original data regarding surgical management and/or survival among patients with pancreatic adenocarcinoma, from inception to January 7th 2017. Our main outcome was the incremental benefit of EUS for the identification of unresectable disease (IB_EUS_). The pooled IB_EUS_ were calculated using random effects models. Heterogeneity was explored using stratified meta-analysis and meta-regression.

**Results:**

Among 4,903 citations identified, we included 8 cohort studies (study periods from 1992 to 2007) that examined the identification of unresectable disease (n = 795). Random effects meta-analysis suggested that EUS alone identified unresectable disease in 19% of patients (95% confidence interval [CI], 10–33%). Among those studies that considered portal or mesenteric vein invasion as potentially resectable, EUS alone was able to identify unresectable disease in 14% of patients (95% CI 8–24%) after a CT scan was performed.

**Limitations:**

The majority of the included studies were retrospective.

**Conclusions:**

EUS evaluation is associated with increased identification of unresectable disease among adults with pancreatic adenocarcinoma.

## Introduction

Pancreatic adenocarcinoma is the fourth leading cause of cancer death in North America, with over 53,000 incident cases expected in 2015.[1] Despite advancement in imaging and surgical techniques, the prognosis remains poor, with an overall 5-year survival of 4–6%.[2] The only potential for cure is targeted surgical resection. Unfortunately, less than 25% of patients have resectable disease at the time of diagnosis.[3, 4]

Current clinical practice guidelines suggest careful preoperative evaluation of all pancreatic adenocarinoma patients to determine resectability status.[5] In addition to focusing surgical resources on those likely to have the greatest benefit, identifying patients with unresectable disease spares the risks associated with surgery and directs care towards more appropriate palliative therapies, including biliary stenting and chemoradiation.

Although multiple preoperative staging techniques have been used in an attempt to accurately stage pancreatic adenocarinoma, the optimal approach remains controversial. There is general agreement that computed tomography (CT) scanning should be the first imaging modality as it is widely available and has excellent sensitivity for identifying resectable disease.[5, 6] Unfortunately, the specificity of this modality for resectability is limited.[6]

Endoscopic ultrasound (EUS) is a safe and highly accurate technique for the detection and staging of pancreatic adenocarcinoma, with a sensitivity comparable to CT scan for identifying resectability[6, 7] and a higher sensitivity than CT scan for detecting nodal involvement and vascular invasion.[6] In a recent meta-analysis, Tamburrino and colleagues [8] demonstrated that EUS is 0.87 sensitive and 0.80 specific for identifying unresectable disease in patient who were believed to have resectable pancreatic adenocarcinoma after a CT scan was performed.

Although the diagnostic accuracy investigations such as EUS aids in appreciating their potential, determining their clinical value requires them to be examined within a diagnostic pathway and evaluated based on their ability to change patient management and improve outcomes.[9] For example, in a recent meta-analysis by James and colleagues [10] preoperative EUS was found to detect pancreatic neuroendocrine tumours in 26% of patients after both CT scan and MRI examinations failed to identify the lesion.

EUS has the potential to influence the surgical management of pancreatic adenocarinoma by identifying patients with locally advanced disease who would not benefit from curative resection. In addition to helping avoid the morbidity and mortality related to unnecessary surgery, identifying patients with unresectable disease may reduce their delay to more beneficial treatments such as chemotherapy.[11,12] Multiple single-centre studies have assessed the impact of EUS on the evaluation and management of pancreatic adenocarcinoma in clinical practice, with varying protocols, outcomes and results. For this reason, we aimed to summarize the literature and present a meta-analysis to determine the pooled incremental benefit of EUS for the identification of unresectable pancreatic adenocarcinoma after a CT scan has been performed.

## Patients and methods

This study was conducted with strict adherence to a detailed protocol created *a priori* in accordance with guidelines for systematic reviews and meta-analyses of observational studies.[13]

### Search strategy

The review approach we used has previously been described.[10] Two investigators (PDJ, ZWM) created an initial electronic bibliographic database search strategy, which was subsequently refined by a medical librarian with extensive systematic review experience. Unrestricted searches of MEDLINE and EMBASE were conducted from their first available date for studies reporting on the association between EUS, surgical decision-making and/or survival in patients with pancreatic adenocarcinoma (the inception search performed on January 18^th^ 2013; with updated searches were performed on December 18^th^ 2014, October 15^th^ 2015 and January 7^th^ 2017). We also manually searched reference lists of all identified relevant publications, reviewed abstracts of conference proceedings (the American College of Gastroenterology, the American Gastroenterology Association, the American College of Surgeons and the Canadian Association of Gastroenterology meetings over the past 3 years) and contacted relevant field experts. Articles published in all languages were considered.[14]

In MEDLINE, we created four comprehensive search themes. The first theme, *endoscopic ultrasound*, combined exploded versions of the Medical Subject Heading (MeSH) terms endoscopic ultrasound-guided fine needle aspiration or endosonography. The second theme, *pancreatic neoplasms*, combined exploded versions of the MeSH terms pancreatic cyst, mucinous and serous neoplasms, carcinoma in situ, pancreatic ductal carcinoma, papillary carcinoma, papillary adenocarcinoma, mucinous adenocarcinoma, mucinous cystadenocarcinoma, noninfiltrating intraductal carcinoma, bile duct neoplasms, acinar cell carcinoma, neuroendocrine tumors, endocrine gland neoplasms, islet cell adenoma, gastrinoma, VIPoma, and glucagonoma. This theme was intentionally broad to identify all studies where cases of pancreatic adenocarcinoma were reported. The third theme, *surgery*, combined exploded versions of the MeSH terms operative surgical procedures, pancreatectomy, laparotomy, pancreaticoduodenectomy. The *endoscopic ultrasound*, *pancreatic neoplasms* and *surgery* search themes were subsequently combined in turn using the Boolean operator “and”.

### Study selection

Three reviewers (PDJ, MZ) independently reviewed titles and abstracts to identify articles that appeared to report original data regarding the ability of EUS to identify unresectable disease. The same reviewers subsequently read all of these articles independently and in full. We used the following inclusion criteria: 1) the study population consisted of adult patients ≥18 years with pancreatic adenocarcinoma; 2) the intervention was preoperative EUS evaluation with or without FNA; and 3) the comparator was exposure to CT scan with or without other imaging modalities, including magnetic resonance imaging (MRI), positron emission tomography (PET) scan and abdominal ultrasound (US). Both published and unpublished studies, including detailed conference abstracts, were eligible for inclusion. Studies with insufficient data or description of the comparator group were excluded. Disagreements regarding article inclusion were resolved by consensus among four of the authors (PDJ, ZWM, MZ, and SJH).

### Data extraction and study outcome

Two reviewers (PDJ, ZWM) independently extracted data from studies fulfilling the inclusion criteria, with any disagreements being resolved by consensus. Data extracted included study setting, study design, sample size, and population demographics. The identification of unresectable disease by EUS alone was measured by calculating the *incremental benefit of EUS* (IB_EUS_), expressed as:

$${IB}_{EUS}=\frac{B_{EUS}}{N_{CT}}$$

**Fig 1:**
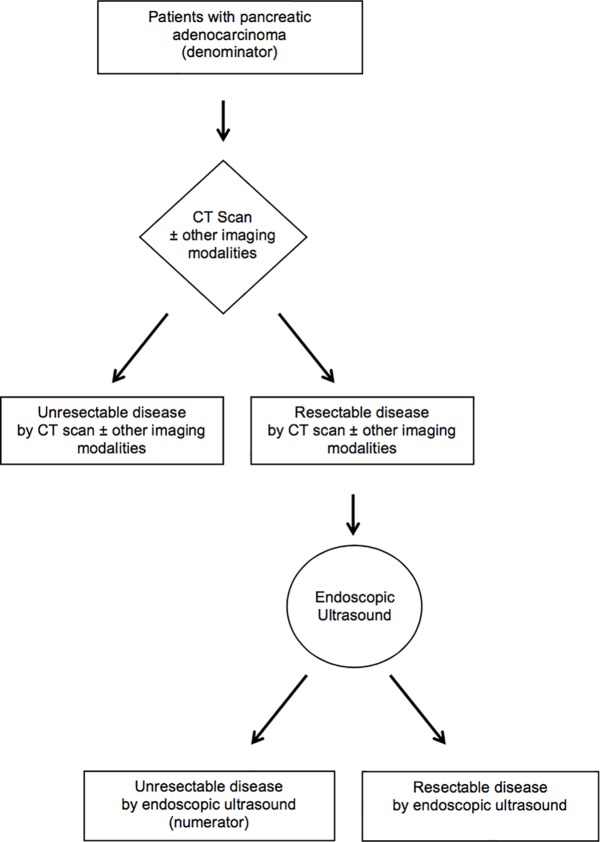
Algorithm applied to calculate the incremental benefit of endoscopic ultrasound (IB_EUS_) for the identification of unresectable disease among patients with pancreatic adenocarcinoma.

Where *N*_*CT*_ is the total number of patients with pancreatic adenocarcinoma who underwent a CT scan and *B*_*EUS*_ is the number of patients who underwent a CT scan and EUS, and the presence of unresectable disease was identified on EUS alone (Fig 1). This outcome is designed to capture the net benefit of EUS above that of CT with or without use of other staging modalities. In situations where other modalities were also used, we determined the number of cases where unresectable disease was detected only by EUS.[10]

### Risk of bias

The same two reviewers also extracted information concerning indicators of study quality. We elected to rate the quality of included studies using the Newcastle-Ottawa Quality Assessment Scale Criteria (http://www.ohri.ca/programs/clinical_epidemiology/oxford.asp). We also evaluated whether or not the included studies a) recruited participants consecutively, b) blinded endosonographers to the results of previous imaging, c) described the type of CT scan used in the preoperative evaluation, d) reported surgical resectability criteria, e) followed the patients to surgery or f) noted potential confounders that could influence surgical management.

## Statistical analyses

We began by calculating the logit of the proportion (*P*) representing the IB_EUS_ as well as its variance. This variance has previously been shown to follow a log-normal distribution and to be precise for proportions greater than 0.8 and less than 0.2.[15] The logit of the proportion (*lp*) was calculated as: log[*P*/(1-*P*)]. The standard error (SE) of *lp* was derived using the equation: √[1/(*P* x Sample Size)/(1/((1-*P*) x Sample Size)).[15] We compared this approach to pooling the *P* using a simplified SE calculation that assumes that *P* follows a binomial rather than log-normal distribution and has a SE = 1/√[*P*/(1-*P*)] and found no difference in summarized results.[15] For sample-size proportional weighting, we calculated the standard error (SE) of each study using the equation: SE = 1/(√sample size).[15]

The lp was summarized across studies using a random effects model and the methods proposed by DerSimonian and Laird.[16] The lp was then converted to the IB_EUS_, as well as a corresponding 95% confidence interval (CI). Small study effects were evaluated through visual inspection of funnel plots and Begg’s asymmetry test.

To assess for heterogeneity of the IB_EUS_ across studies, we inspected forest plots for asymmetry and calculated I^2^ inconsistency statistics. We conducted stratified meta-analyses and meta-regression to evaluate the influence of study-level characteristics on the pooled estimates of effect. *A priori* characteristics of interest included study region, comparator group, design, quality and follow-up.

To ascertain a range for the IB_EUS_, we developed two scenarios *a priori* that could considerably increase or decrease the proportion of cases where EUS alone identified unresectable disease. In the first case, we only considered studies where CT scan was the sole imaging modality performed prior to EUS and all the patients in the cohort were exposed to EUS. This could increase the estimate of effect. In the second scenario, we assumed that all cases with portal or mesenteric vein invasion (PMVI) were resectable.[5] We excluded studies that did not specify the number of cases related to vascular invasion. For cases with PMVI, only the presence of local invasion, ascites, metastatic tumour deposition or lymphadenopathy was used to determine resectability status. All statistical analyses were performed using Stata/IC version 11.1 (StataCorp, College Station, TX, USA).

## Results

### Identification of articles

**Fig 2:**
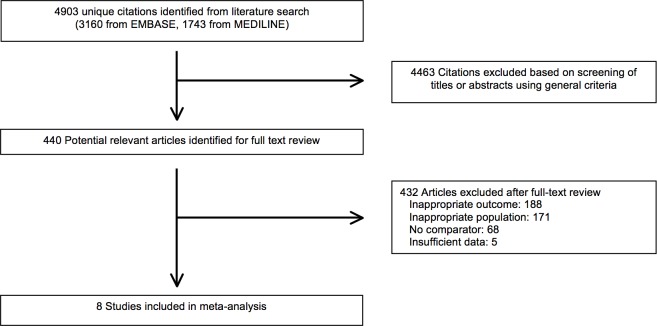
PRISMA flow-chart of included studies for meta-analysis.

The flow of the articles through the systematic review is summarized in Fig 2. Among 4,903 unique citations identified, we included 8 in the systematic review. These articles reported on the association between EUS and the identification of unresectable disease. Inter-rater agreement for the abstract review was moderate (k = 0.61, 95% CI 0.58–0.64).

### Study characteristics

Characteristics of the 8 included studies are shown in Table 1. The study period for included studies was 1992 to 2007. [17–24] All of these were single-center studies and four were performed in North America. Two were prospective and six were retrospective. Among all studies, the number of enrolled participants ranged from 24 to 411, for a total of 1,030 participants across all studies. Out of the 1,030 participants, 795 fulfilled the criteria to be included in the meta-analysis. All of these studies included CT scan as a comparator. Two studies [18, 23] compared EUS-based staging with surgical staging (combined total of 10 patients) and they were the same in all cases.

**Table 1:** Characteristics of studies included for the meta-analysis.

Study	Study Period	Comparator	Cohort Designation	Country	Total No. of Patients	No. of Patients Included*	Average Age (years)
Suits et al, 1999[17]	1994–1998	CT	Prospective	USA	98	98	67
Queneau et al, 2001[18]	1995–1999	CT/US	Restrospective and Prospective	France	64	64	71
Mortensen et al, 2001[19]	1997–1999	CT	Restrospective	Denmark	101	99	65
Fristrup et al, 2006[20]	2002–2004	CT	Unclear	Denmark	179	146	66**
Kliment et al, 2010[21]	2007–2007	CT/MRI/US	Restrospective	Czech Republic	213	152	62
Croome et al, 2010[22]	2005–2006	CT	Restrospective	Canada	133	133	Missing
Cahn et al, 1996[23]	1993–1995	CT	Unclear	USA	50	24	60
Buscail et al, 1999[24]	1992–1997	CT	Prospective	USA	79	79	67

CT = Computed tomography; US = Abdominal ultrasound; MRI = Magnetic resonance imaging.

EUS = Endoscopic ultrasound.

*Number of patients included in the meta-analysis.

**Median age.

### Risk of bias assessment

Table 2 presents the presence/absence of a number of key indicators of study quality, for each of the studies included in our review, along with summary scores from the Newcastle-Ottawa Quality Assessment Scale. The average quality score from this scale was 7.6 out of 9. Only one study reported blinding to previous imaging performed prior to the EUS evaluation. Consecutive recruitment was noted in five studies. Five of eight included studies examining disease resectability did not describe the type of CT scan used.

**Table 2:** Quality assessment of included studies.

Study	Study Setting	Cohort Described	Consecutive RecruitmentDescribed	Type of CT Scan Described	EUS Exposed to All Patients	Confounders Discussed*	Blinding to Previous Imaging	Criteria for Resectability Described	Tumour Size or Location Described	Quality Assessment**
Suits et al, 1999[17]	Single-center	Yes	No	Yes	Yes	No	No	Yes	No	7
Queneau et al, 2001[18]	Single-center	Yes	Yes	Yes	Yes	No	No	Yes	No	8
Mortensen et al, 2001[19]	Single-center	Yes	No	No	Yes	No	No	No	No	7
Fristrup et al, 2006[20]	Single-center	Yes	Yes	No	Yes	No	No	Yes	No	7
Kliment et al, 2010[21]	Single-center	Yes	Yes	No	Yes	Yes	No	No	Yes	8
Croome et al, 2010[22]	Single-center	Yes	No	No	No	Yes	No	No	Yes	8
Cahn et al, 1996[23]	Single-center	Yes	Yes	No	Yes	Yes	No	Yes	Yes	8
Buscail et al, 1999[24]	Single-center	Yes	No	Yes	Yes	Yes	No	Yes	Yes	8

*Potential confounders that could have also influenced surgical management include patient age, comorbidity and preferences.

** Evaluated using the Newcastle-Ottawa Quality Assessment Scale (maximum 9 stars).

### Proportion of cases where EUS alone identified unresectable disease

**Fig 3:**
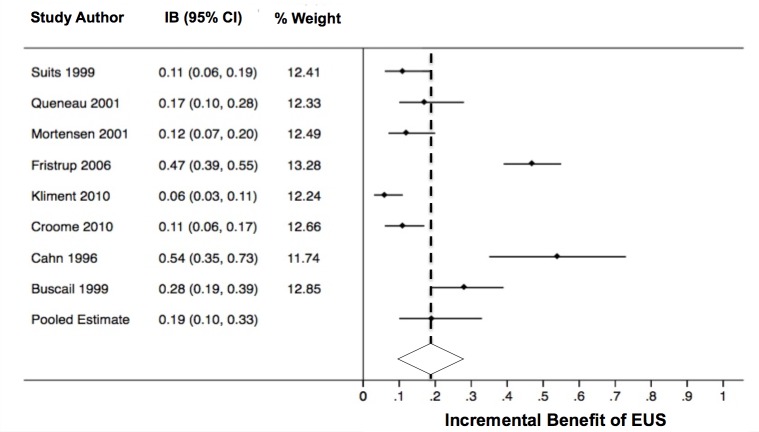
The incremental benefit of endoscopic ultrasound for the identification of unresectable disease. IB_EUS_ = Incremental benefit of endoscopic ultrasound; CI = Confidence interval; EUS = Endoscopic ultrasound; I^2^ = 84.8%, *P*<0.0001

The pooled IB_EUS_ was 19% (95% CI 10–33%, see Fig 3). There was significant heterogeneity noted across studies (I^2^ = 93.0%, *P*<0.001). When exploring heterogeneity using stratified analyses across key study characteristics (Table 3), study-level factors that appeared to influence the pooled IB_EUS_ estimate included CT scan type (one study noted a spiral CT scan was used and this study had a lower IB_EUS_), the use of other imaging modalities (one study included MRI and US in addition to CT scan and EUS and this study showed a lower IB_EUS_), and description of resectability criteria (six studies explicitly described their resectability criteria and the pooled IB_EUS_ was greater in this group).

**Table 3:** Stratified analysis of pooled incremental benefit of endoscopic ultrasound for the identification of unresectable disease.

Stratified Analysis	Number of Studies	Pooled Proportion (IB_EUS_)	Heterogeneity I^2^ Statistics (%)	*P* Value	
				I^2^ Statistics	Meta-regression
**Study Type**					
** Prospective**	2	0.18 (0.07, 0.4)	86.7%	<0.01	0.048
** Retrospective**	3	0.09 (0.06, 0.14)	36.8%	0.205	
** Both**	1	0.17 (0.10, 0.28)	NA	N/A	
** Not described**	2	0.48 (0.40, 0.55)	0.0%	0.49	
**Study Period**					
** After 2000**	3	0.16 (0.03, 0.51)	97.1%	<0.01	0.990
** Before 2000**	5	0.21 (0.12, 0.36)	84.6%	<0.01	
**Study Location**					
** Within North America**	4	0.22 (0.1, 0.42)	89.7%	<0.01	0.966
** Outside of North America**	4	0.17 (0.05, 0.42)	95.5%	<0.01	
**Comparators**					
** CT, Ultrasound and MRI**	1	0.06 (0.03, 0.11)	NA	NA	0.477
** CT and Ultrasound**	1	0.17 (0.10, 0.28)	NA	NA	
** CT Alone**	6	0.23 (0.12, 0.41)	93.1%	<0.01	
**CT type**					
** Conventional**	2	0.18 (0.07, 0.4)	86.7%	<0.01	0.793
** Spiral**	1	0.17 (0.10, 0.28)	NA	NA	
** Unclear**	5	0.20 (0.07, 0.45)	95.5%	<0.01	
**Tumor Location or Size Mentioned**					
** Yes**	4	0.19 (0.07, 0.42)	92.5%	<0.01	0.794
** No**	4	0.19 (0.07, 0.42)	94.1%	<0.01	
**Resectability Criteria Noted**					
** Yes**	6	0.23 (0.11, 0.41)	93.8%	<0.01	0.391
** No**	2	0.12 (0.08, 0.17)	0.0%	0.85	
**Clinical factors Discussed**					
** Yes**	5	0.19 (0.09, 0.36)	90.0%	<0.01	0.684
** No**	3	0.20 (0.06, 0.51)	95.7%	<0.01	
**Quality Score**					
** ≥8 stars**	5	0.19 (0.09, 0.36)	90.0%	<0.01	0.684
** 7 stars**	3	0.20 (0.06, 0.51)	95.7%	<0.01	
**EUS Exposure All Patients**					
** Yes**	7	0.21 (0.11, 0.37)	93.0%	<0.01	0.510
** No**	1	0.11 (0.06, 0.17)	NA	NA	

IB_EUS_ = Incremental benefit of endoscopic ultrasound.

When considering studies where CT scan was the only other imaging modality performed and EUS was used among all patients, the pooled IB_EUS_ was 23% (95% CI 12–41%). In contrast, when excluding studies that did not describe vascular invasion and classifying cases with PMVI alone as resectable, the pooled IB_EUS_ decreased to 14% (95% CI 8–24%).

Of note, seven studies presented adequate data to describe the reason identifying the cancer as unresectable (total n = 662). The reasons noted include spread to local organs (n = 43, 6%), lymph node involvement (n = 28, 4%), arterial (celiac or superior mesenteric artery) invasion (n = 11, 2%), distant metastases (n = 16, 2%), malignant ascities (n = 1, 0.2%), and venous (portal, splenic or superior mesenteric vein) occlusion (n = 18, 3%). Four studies described the use of FNA during the pancreatic staging evaluation (total n = 373). Overall, FNA was performed in 232 patients (62%). Two of studies used FNA for all the patients who underwent a EUS, while FNA was used in 11% and 50% of the patients in the other investigations, respectively.

### Assessment for evidence of publication bias

For the studies examining the identification of unresectable disease, visual inspection of the funnel plot did not show asymmetry, suggesting no significant publication bias. This was confirmed with Begg’s test (*P* = 0.14; S2 Fig).

## Discussion

In this systematic review and meta-analysis, we observed that preoperative EUS evaluation increases the detection of unresectable disease among patients with pancreatic adenocarcinoma. EUS detected advanced disease in up to 19% of patients with pancreatic adenocarcinoma that were deemed resectable by CT scan. This would translate to a number needed to test of approximately five to avoid one non-beneficial, costly and potentially harmful surgery among patients with pancreatic adenocarcinoma.

Stratified and sensitivity analyses revealed multiple clinically meaningful relationships. The use of multiple imaging modalities prior to EUS reduces the potential utility of EUS. This was especially true when both a CT and MRI are performed prior to EUS. Although MRI may also inform appropriate surgical management, EUS may be more accurate in determining resectability and can also be used to obtain a definitive tissue diagnosis through fine needle aspiration.[25]

Studies examining conventional CT presented a greater incremental benefit for EUS compared to the one more recent study that used spiral CT. The use of newer CT scan technology such as thin-slice and multi-detector CT (MDCT) scanners could result in fewer cases of unresectable disease being recognized by EUS alone, however this has not been demonstrated to date. EUS has been shown to be more accurate at detecting and staging pancreatic cancer compared to MDCT.[26–28] EUS is unable to reliably identify distant metastases, however, and should be viewed as an adjunct to compliment CT scan evaluation. Further study regarding incremental benefit of preoperative EUS after evaluation using newer CT technologies is warranted.

Recent consensus statements and guidelines note that cases with limited portal vein, mesenteric vein or superior mesenteric artery invasion may be suitable for safe resection.[5] The IB_EUS_ was reduced to 14% when the presence of PMVI alone was considered resectable disease. This results in a number needed to test of seven to avoid one futile surgery. EUS has recently been shown to have a sensitivity superior to CT scan for detecting vascular invasion[6] and can play important role in characterizing the extent of vascular invasion to support surgical management as well as neo-adjuvant therapy. Innovative EUS techniques, such as contrast-enhanced harmonic and three-dimensional EUS, may enhance the accuracy of local cancer staging by EUS [29] thereby potentially increasing incremental benefit of this modality.

We elected to use the identification of unresectable disease by EUS alone as our main outcome of interest. This was done to define a hard outcome that can be examined across multiple studies. EUS, however, can play multiple roles to support the surgical and medical management of patients with pancreatic adenocarinoma. These include the detection of small pancreatic cancers missed on previous imaging and EUS-guided FNA for diagnostic confirmation. Multiple investigators have recommended that EUS be performed on all patients with CT evidence pancreatic adenocarcinoma.[30, 31] New therapeutic roles of EUS are emerging, such as FNA sampling of malignant tissue to tailor adjuvant therapy, EUS-guided fiducial marker placement for image-guided radiation therapy and EUS-guided brachytherapy.

There are several limitations of this meta-analysis that should be considered. First, the quality of the individual studies was limited. The most noteworthy limitation is that the majority of the studies we examined were retrospective and therefore prone to bias. Second, the majority of studies we summarized do not describe the type of CT scan used. Third, there was significant heterogeneity among the studies that examined median survival. This may reflect the small sample sizes and design diversity among the included studies. For this reason, we elected to use a random effects model for pooled estimates. We also explored for sources of heterogeneity using stratified analyses, which allowed us to describe how varying study characteristics influenced the pooled estimate. These are key components to performing meta-analyses when significant heterogeneity is identified.[15, 16] Fourth, the funnel plot may not be able detect small study or publication bias when only a small number of studies are considered.[32] Nevertheless, it is reassuring to see that there was good funnel plot symmetry and Begg’s test was negative. Finally, multiple factors not measured in the included studies can influence pancreatic cancer resectability, including patient comorbidities and preferences. The inability to identify and adjust for confounders is an important limitation of observational studies that could only be addressed using a randomized control trial design.

In conclusion, this systematic review demonstrates that preoperative EUS evaluation is associated with an increased identification of unresectable disease patients with pancreatic adenocarcinoma. Identifying advanced disease and limiting non-beneficial surgical resections may be one of the mechanisms by which EUS may have a positive effect on patient care.

## Supporting information

S1 Fig PRISMA checklist. (PDF)

S2 Fig Funnel plot for studies considering the identification of unresectable disease. The pseudo 95% confidence interval (CI) corresponds to the expected 95% CI for a given standard error.
(TIFF)

## Abbreviations

CT: Computed tomography
EUS: Endoscopic ultrasound
FNA: Fine-needle aspiration
IB_EUS_: Incremental proportional of endoscopic ultrasound
MDCT: Multi-detector computed tomography
MeSH: Medical subject heading
MRI: Magnetic resonance imaging
MSR: Median survival ratio
PET: Positron emission tomography
PMVI: Portal or mesenteric vein invasion
US: Abdominal ultrasound
