# Supplementary figures and images for: The incremental benefit of EUS for identifying unresectable disease among adults with pancreatic adenocarcinoma: A meta-analysis
Source: PLoS One. 2017 Mar 20;12(3):e0173687. doi: 10.1371/journal.pone.0173687 (PMC5358870; doi:10.1371/journal.pone.0173687)

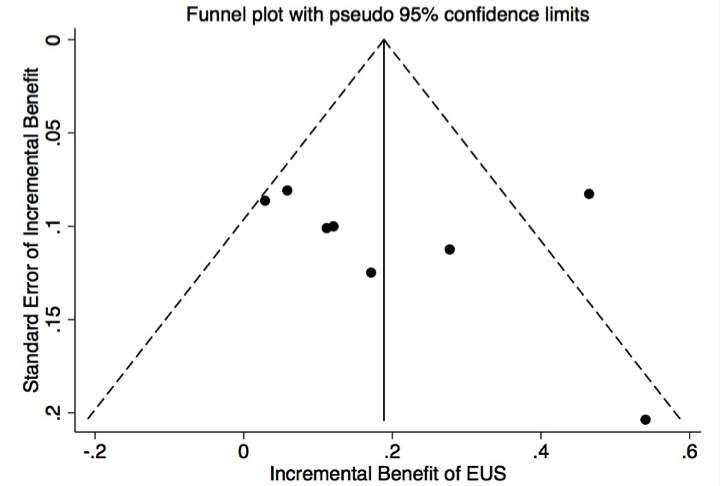

Supplement: S2 Fig — The pseudo 95% confidence interval (CI) corresponds to the expected 95% CI for a given standard error. (TIFF) [file pone.0173687.s002.tiff]
